# Effect of autologous concentrated growth factor on bedsore wounds in elderly patients with diabetes: a case-control study

**DOI:** 10.3389/fendo.2025.1620730

**Published:** 2025-07-18

**Authors:** Qin-wen Bao, Jing Xu, Zhi Wang, Guangyun Hu, Wen Zhong, Yunfeng Li, Xiao-lei Dong, Tong-dao Xu, Chong Gao

**Affiliations:** ^1^ Department of Geriatrics, Lianyungang Second People’s Hospital Affiliated to Kangda College, Nanjing Medical University, Lianyungang, Jiangsu, China; ^2^ Department of Oncology, Lianyungang Second People’s Hospital Affiliated to Kangda College, Nanjing Medical University, Lianyungang, Jiangsu, China; ^3^ Department of Endocrinology, The Second People’s Hospital of Lianyungang, Lianyungang, Jiangsu, China; ^4^ Department of Orthopedics, Affiliated Lianyungang Clinical College of Nantong University, Lianyungang, Jiangsu, China

**Keywords:** autologous concentrated growth factor, pressure ulcers, elderly patients, diabetes, wound healing, regenerative medicine

## Abstract

**Background:**

Pressure ulcers, also known as bedsores, are common injuries to the skin and subcutaneous tissues in patients who are bedridden or wheelchair-bound, with a particularly high incidence among elderly patients with diabetes. These chronic wounds often lead to increased morbidity, prolonged hospitalization, and reduced quality of life. Traditional treatments for pressure ulcers have limited efficacy. In recent years, autologous concentrated growth factor (ACGF) has emerged as a promising regenerative medicine approach, showing potential in promoting wound healing through enhanced cell proliferation, accelerated angiogenesis, and improved tissue regeneration.

**Objective:**

This study aims to evaluate the effectiveness of ACGF in treating pressure ulcers in elderly diabetic patients by comparing wound healing, symptom relief, and inflammatory markers with those receiving conventional therapy.

**Methods:**

This analysis included 51 elderly patients aged 60 years or older with diabetes and pressure ulcers. Patients were divided into two groups: 26 received standard wound care (Control Group, CG), and 25 received ACGF treatment in addition to standard care (Treatment Group, TG). ACGF was prepared using a standardized protocol and applied directly to the wound site. Pain levels (VAS scores), wound healing (PUSH scores), and inflammatory markers (WBC, CRP, PCT, and IL-6) were compared between the two groups before treatment, 14 days, and 28 days after treatment.

**Results:**

VAS Scores: Before treatment, there was no significant difference between the two groups (TG: 6.92 ± 0.86, CG: 6.69 ± 1.01, P=0.392). At 14 days post-treatment, the VAS scores in the TG were significantly lower than those in the CG (TG: 3.52 ± 0.51, CG: 4.46 ± 0.58, P<0.001). By 28 days, the VAS scores in the TG further decreased (TG: 1.24 ± 0.44, CG: 1.58 ± 0.70, P=0.046). PUSH Scores: Before treatment, there was no significant difference between the two groups (TG: 14.84 ± 1.72, CG: 14.19 ± 1.92, P=0.211). At 14 days, the TG showed a significantly lower PUSH score than the CG (TG: 6.52 ± 0.71, CG: 8.23 ± 0.77, P<0.001). By 28 days, the PUSH scores in the TG continued to decrease (TG: 2.52 ± 0.59, CG: 3.39 ± 0.50, P=0.001). Inflammatory Markers: Before treatment, there were no significant differences in WBC, CRP, PCT, and IL-6 levels between the two groups (P>0.05). At 14 days post-treatment, the TG exhibited significantly lower levels of WBC (TG: 7.44 ± 1.56, CG: 8.60 ± 1.98, P=0.024) and PCT (TG: 0.63 ± 0.45, CG: 1.29 ± 0.48, P<0.01). By 28 days, the TG also showed significant reductions in CRP (TG: 5.93 ± 9.74, CG: 18.63 ± 6.62, P<0.01) and IL-6 (TG: 3.35 ± 1.89, CG: 5.56 ± 2.22, P<0.01).

**Conclusion:**

This study suggests that ACGF is an effective adjunctive treatment for pressure ulcers in elderly diabetic patients. By significantly enhancing wound healing and reducing inflammatory responses, ACGF could serve as a valuable addition to standard care protocols for this vulnerable population. Further prospective studies are warranted to confirm these findings and explore the underlying mechanisms of ACGF in wound healing.

**Clinical Trial Registration:**

https://www.medicalresearch.org.cn, identifier MR-32-24-019758.

## 1 Introduction

Pressure ulcers, also known as bedsores, are common skin and subcutaneous tissue injuries in patients who are bedridden or in wheelchairs, typically occurring at bony prominences (1, 2). The incidence of pressure ulcers among hospitalized patients can reach 10% to 30%, and it is even higher in long-term care institutions (3). Pressure ulcers not only cause significant pain to patients but also greatly reduce their quality of life, prolong hospitalization, and increase medical expenses. With the aging population, the incidence of chronic diseases, especially diabetes, is rising among elderly patients, leading to an increased prevalence of pressure ulcers (2). Diabetic patients are particularly at risk because hyperglycemia leads to microvascular and neuropathic changes, impairing blood supply and sensation in the skin and subcutaneous tissues, reducing their tolerance to pressure and friction, thus significantly increasing the risk of pressure ulcer development (4, 5). Studies have shown that the incidence of pressure ulcers in diabetic patients is 1.5 to 2 times higher than in non-diabetic patients (6). Furthermore, diabetic patients have poor wound healing abilities, making the treatment of pressure ulcers particularly challenging (7). Therefore, effective treatment methods are urgently needed to improve healing outcomes and alleviate patient suffering in diabetic elderly patients with pressure ulcers.

The treatment of pressure ulcers in diabetic patients presents numerous challenges. First, the hyperglycemic environment provides favorable conditions for bacterial growth, significantly increasing the risk of wound infections, which are often difficult to control once they occur (5, 8). Additionally, microvascular damage and neuropathy caused by diabetes further impair skin blood circulation and sensation, delaying wound healing (9, 10). Although traditional treatment methods such as debridement, antibacterial treatment, and local dressings are somewhat effective, they are limited in treating chronic, difficult-to-heal pressure ulcers in diabetic patients (11). For example, debridement may be insufficient due to the poor tissue repair abilities of diabetic patients, making it hard to completely remove necrotic tissue (12). Local dressings also often fail due to excessive exudate caused by hyperosmotic conditions (13). Overall, the treatment of pressure ulcers in diabetic patients requires not only blood sugar control and improved blood circulation but also the resolution of issues such as wound infection and slow tissue repair (14). However, current treatments have significant limitations when addressing these complex problems, and the treatment is much more difficult than handling a single issue.

Therefore, exploring new treatment options to improve the healing of pressure ulcers in diabetic patients is especially urgent. In recent years, with the rapid development of regenerative medicine, autologous concentrated growth factor (ACGF) has gained attention as an emerging biological treatment technology (15). ACGF was first proposed by Sacco in 2006 and applied clinically (16). It is a concentrated autologous blood product obtained using a special centrifuge, which activates α-granules in platelets through physical acceleration and deceleration, producing a product rich in growth factors and CD34+ cells (17–19). ACGF has shown superior regenerative abilities for bone tissue, soft tissue, and skin, and is widely used in fields such as dentistry and plastic surgery (20). ACGF contains various growth factors, such as TGF-β (transforming growth factor-β), PDGF (platelet-derived growth factor), VEGF (vascular endothelial growth factor), IGF (insulin-like growth factor), EGF (epidermal growth factor), and FGF (fibroblast growth factor), which effectively promote tissue regeneration (21). In addition, ACGF is rich in fibrin, which is viscous, with white blood cells, platelets, and growth factors adhering to the surface and inside of the fibrin scaffold, exerting anti-inflammatory effects, releasing growth factors, and other biological effects (22). ACGF has high tensile strength, is easy to shape, and is rich in CD34+ cells, which play an important role in vascular maintenance, regeneration, tissue repair, and immune regulation. Compared to traditional blood concentrate products (such as PRP and PRF), the presence of CD34+ cells is a significant advantage of ACGF (23).

Given the complex pathophysiology of diabetic pressure ulcers and the limitations of traditional treatments, ACGF is expected to provide a new solution to this clinical problem. Therefore, this study aims to explore the clinical efficacy of ACGF in the treatment of pressure ulcers in elderly diabetic patients, by assessing its impact on wound healing, pain relief, and inflammation, to provide new insights and evidence for the treatment of diabetic pressure ulcers.

## 2 Materials and methods

This study was conducted in Lianyungang Second People’s Hospital Affiliated to Kangda College, from January 2022 to December 2024. The study was approved by the institutional review board (IRB) of Lianyungang Second People’s Hospital Affiliated to Kangda College, The Ethics approval number is 2022K047, and all patients signed informed consent forms. The aim was to evaluate the effectiveness of autologous concentrated growth factor (ACGF) in the treatment of pressure ulcers in elderly diabetic patients.

### 2.1 Patient selection

The inclusion criteria comprised:

1. Patients aged 60 years or older.
2. A confirmed diagnosis of diabetes (Type 1 or Type 2).
3. Presence of pressure ulcers classified as grade II or higher according to the National Pressure Injury Advisory Panel (NPIAP) guidelines.
4. Patients who provided informed consent for participation in the study.

Exclusion criteria included:

1. Patients with active infections at the wound sites.
2. Patients receiving systemic corticosteroids or immunosuppressive therapy within the past three months.
3. Patients with coagulopathy or other contraindications for blood product administration.
4. Patients with significant cognitive impairment that prevented informed consent.

**Figure 1:**
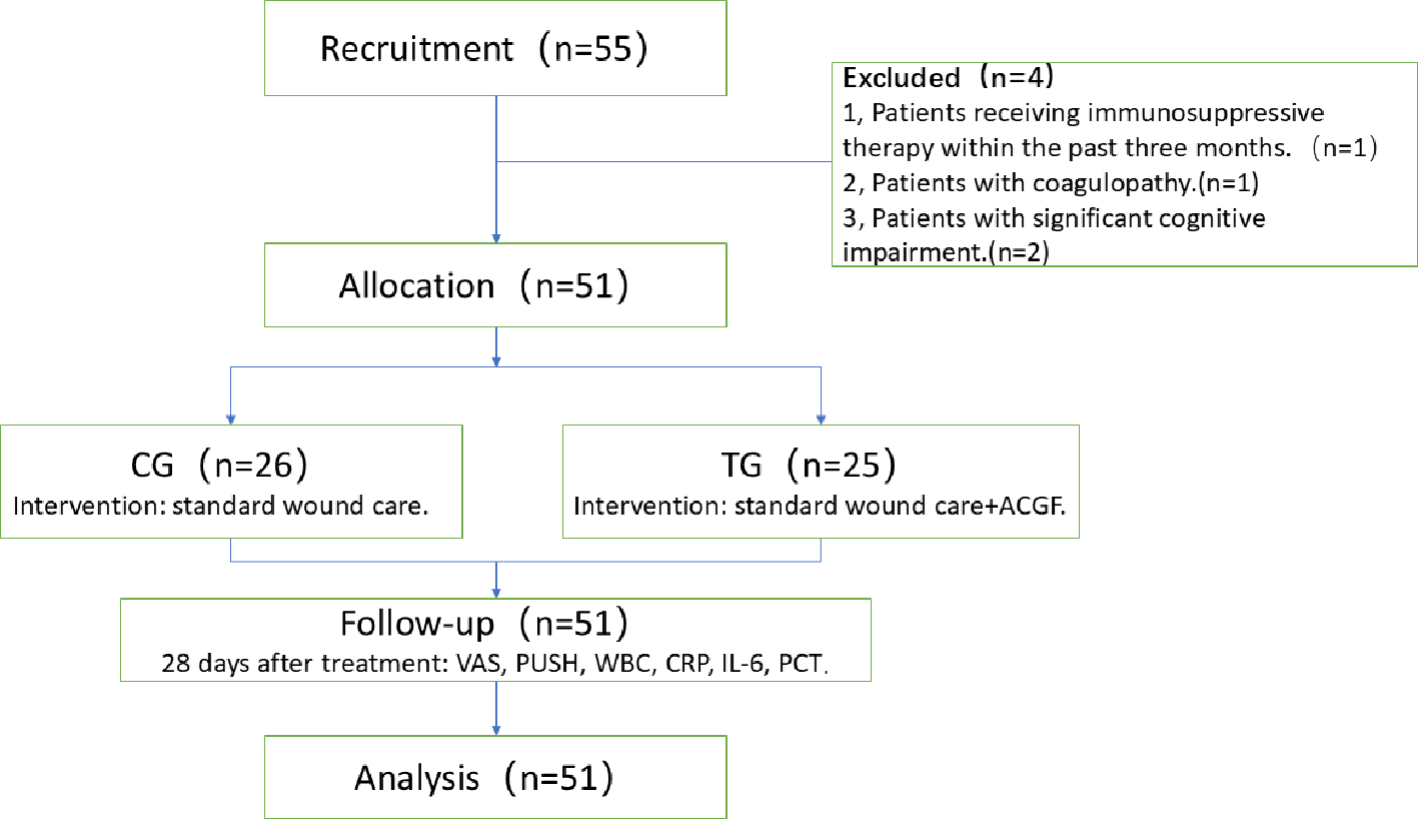
Preparation of ACGF: **(a)** Centrifuge and Tubes: The Medifuge Silfradent centrifuge and specific tubes from Silfradent are used. **(b)** Centrifugation of Blood: After centrifugation, the CGF tube is obtained, Red parentheses indicate the parts related to ACGF. **(c)** The separated ACGF, as indicated by the red arrow.

A total of 55 elderly patients, aged 60 years and older, diagnosed with diabetes mellitus and suffering from grade II or higher grade were included. Four patients were excluded, and a total of 51 patients were enrolled in this study (**Figure 1**).

Patients were divided into two groups: 26 received standard wound care (Control Group, CG), and 25 received ACGF treatment in addition to standard care (Treatment Group, TG). A balance test was performed on the baseline characteristics of the two groups (continuous variables were tested using t-tests or Wilcoxon rank-sum tests, and categorical variables were tested using chi-square tests or Fisher’s exact tests) to ensure that the differences in key variables between the two groups were not statistically significant (P>0.05), thereby enhancing the comparability between the two groups. The CG included 13 males and 13 females; the average age was (78.88 ± 9.06) years; the number of pressure ulcers was 29; the pressure ulcer stages were: II stage 5, III stage 14, and IV stage 10. The TG included 15 males and 10 females; the average age was (73.88 ± 10.92) years; the number of pressure ulcers was 26; the pressure ulcer stages were: II stage 3, III stage 9, and IV stage 14. Comorbidities assessed by the Charlson score (24) include cerebrovascular disease, cardiovascular disease, renal impairment, etc. The nutrition score was assessed according to the Mini Nutritional Assessment-Short Form(MNA-SF) (25). There were no significant differences in gender, age, pressure ulcer stages, comorbidities and nutrition score between the two groups (P>0.05), They are comparable (**Table 1**).

**Table 1:** Comparison of the general data between the two patient groups.

Groups	Example number	Gender (male/female)	Age	Pressure ulcer stages (II/III/IV)	Charlson score	MNA-SF
TG	25	15/10	73.88 ± 10.92	3/9/14	5.48 ± 1.74	10.80 ± 1.73
CG	26	13/13	78.88 ± 9.06	5/14/10	5.00 ± 1.23	10.15 ± 1.71
*T／χ２*		0.515	-1.784	2.096	-1.238	1.339
*P*		0.473	0.081	0.351	0.222	0.187

### 2.2 Treatment protocol

A highly individualized hypoglycemic plan should be developed by comprehensively considering multiple factors of geriatric patients, such as cardiac function, liver and kidney function, complications and comorbidities, hypoglycemia risk, frailty state, body weight, and the preferences of patients and their families. The glycemic control targets are as follows: HbA1c ≤ 8.5%, fasting blood glucose ≤ 8.5 mmol/L, and 2 - hour postprandial blood glucose < 13.9 mmol/L. Priority should be given to using metformin and other medications with a low risk of hypoglycemia. For elderly T2DM patients with atherosclerotic cardiovascular disease (ASCVD) or high - risk factors, SGLT2 inhibitors (SGLT2i) or glucagon - like peptide - 1 receptor agonists (GLP - 1RA) with evidence of ASCVD benefits should be the first choice. In elderly T2DM patients with heart failure or chronic kidney disease (CKD), SGLT2i should be preferred. For elderly T2DM patients with CKD who cannot tolerate SGLT2i, GLP - 1RA with evidence of CKD benefits can be chosen. For elderly patients with severe conditions or poor response to oral medications, long - acting basal insulin should be used, and blood glucose levels should be regularly monitored before and 2 hours after each meal. Treatment strategies should be adjusted in a timely manner based on blood glucose changes.

The systolic blood pressure control target for elderly diabetic patients included in this study is below 150 mmHg to reduce the risk of cardiovascular diseases. Blood pressure should be closely monitored to prevent orthostatic hypotension, postprandial hypotension, and excessively low diastolic pressure. Antihypertensive drugs of choice are ACE inhibitors (ACEI) or angiotensin receptor blockers (ARB), with monitoring of serum potassium and creatinine levels. If blood pressure cannot be controlled with ACEI or ARB monotherapy, calcium channel blockers, thiazide diuretics, or β - blockers can be added to enhance the antihypertensive effect. Low - density lipoprotein cholesterol (LDL - C) should be controlled below 2.6 mmol/L using statin therapy. For patients with diabetic nephropathy, a daily intake of high - quality protein at 0.8 g/kg is recommended, along with sodium restriction. Consuming < 5 g/d of sodium chloride or < 2 g/d of sodium helps lower blood pressure and the risk of cardiovascular diseases. Multidisciplinary comprehensive management of elderly diabetic patients with CKD should be carried out in collaboration with nephrologists.

Patients were divided into two groups:

CG: Received standard wound care: Assess the patient’s overall condition and evaluate the wound. Perform surgical debridement, prepare the wound bed, use sensitive antibiotics for prevention, apply specialized support surfaces for pressure offloading, and dress the wound with sterile dressings.
TG: In addition to standard wound care, the TG group underwent ACGF therapy for pressure ulcer treatment.

**Figure 2:**
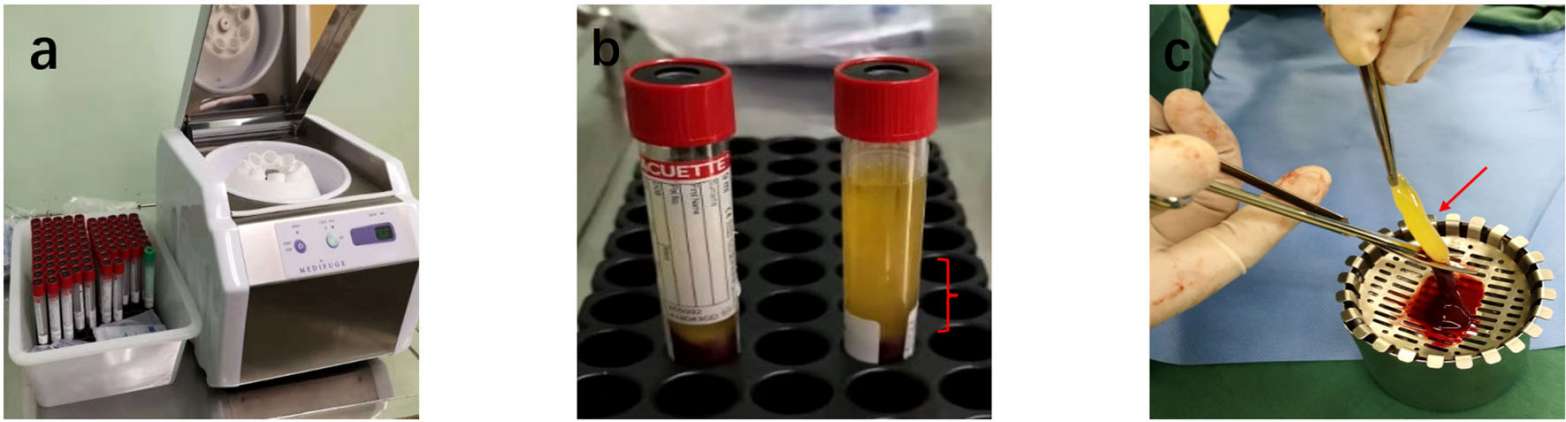
Preparation of ACGF: **(a)** Centrifuge and Tubes: The Medifuge Silfradent centrifuge and specific tubes from Silfradent are used. **(b)** Centrifugation of Blood: After centrifugation, the CGF tube is obtained, Red parentheses indicate the parts related to ACGF. **(c)** The separated ACGF, as indicated by the red arrow.

Preparation of Autologous Concentrated Growth Factor (ACGF): ACGF was prepared according to a standardized protocol. The centrifugation equipment used was a Mediuge concentrated growth factor from Italian company Salfadent and Zihe fibrinogen centrifugal manufacturing machine. Using a CGF-specific centrifuge tube Mediuge 9 ml vacuum centrifuge tube, the blood collection tube was produced by Greiner from Austria Bio-One Manufacturing. The process involved:

1. Blood Collection: Approximately 20–30 mL of venous blood was drawn from each patient into sterile tubes containing an anticoagulant (EDTA or sodium citrate).
2. Centrifugation: Blood samples were automatically continuously centrifuged with variable speed in four stages, first accelerated for 30 seconds, centrifuged at 2,700 rpm for 2 minutes, then at 2,400 rpm for 4 minutes; centrifuged at 2,700 rpm for 4 minutes; centrifuged at 3,000 rpm for 3 minutes, and finally decelerated for 36 seconds to stop, using a centrifuge(The MediFuge CGF centrifuge manufactured by Sefadent, Italy). This step separated the blood components into three layers: red blood cells at the bottom, a middle layer containing concentrated growth factors (the buffy coat), and plasma at the top.
3. Extraction of Growth Factors: The buffy coat was carefully extracted using a sterile syringe and transferred to a separate sterile container for application (**Figure 2**).
4. Application: ACGF was applied directly to the wound bed during each dressing change, typically once a week.

### 2.3 Observation indicators and data collection

All of the observation indicators were measured at three stages: before treatment, 14 days after treatment, and 28 days after treatment.

#### 2.3.1 VAS score

Evaluate the pain level of two groups using the VAS score, with a maximum score of 10 points. The higher the score, the more severe the pai (26). The VAS was administered as a 10-cm written horizontal line, with endpoints labeled “0 = no pain, 1 = slight pain, 3 = mild pain, 5 = moderate pain, 7 = severe pain, 10 = worst possible pain”. Participants were instructed to mark the line corresponding to their pain level, with research assistants blinded to the study hypothesis.

#### 2.3.2 PUSH score

Evaluate the recovery of two groups of pressure ulcer wounds using the Pressure Ulcer Healing (PUSH) score, based on wound area, exudate, and wound type Assessment, with a total score of 0–17 points, where 0 points indicate healing of pressure ulcers, and the smaller the score, the better the recovery effect of pressure ulcer wounds. Wound assessments were performed by trained wound care nurses blinded to the group assignments. Measurements included:

1. Wound Size: The area of the wound was calculated using the formula for the area of an ellipse (length × width × π/4). The length: The longest linear distance between two points on the wound margin (regardless of wound contour) and the width: The longest perpendicular distance to the defined length, measured at the widest point of the wound. 0 points for area 0 cm^2^, 1 points for area <0.3 cm^2^, 2 points for area 0.3~0.6 cm^2^, 3 points for area 0.7~1.0 cm^2^, 4 points for area 1.1~2.0 cm^2^, 5 points for area 2.1~3.0 cm^2^, 6 points for area 3.1~4.0 cm^2^, 7 points for area 4.1~8.0 cm^2^, 8 points for area 8.1~12.0 cm^2^, 9 points for area 12.1~24.0^2^, 10 points for area > 24.0cm^2^.
2. Amount of Exudate: 0 points for no exudate; 1 point for a small amount of exudate; 2 points for a moderate amount of exudate; 3 points for a large amount of exudate.
3. Tissue Type: 0 points for intact or non-broken skin; 1 point for a superficial ulcer without slough or eschar; 2 points for an ulcer with slough but without eschar; 3 points for an ulcer with eschar.

#### 2.3.3 Inflammatory markers

Including white blood cell count(WBC), c-reactive protein(CRP), procalcitonin(PCT), and interleukin 6. Blood sampling should be preceded by an 8-hour fasting period. 5 ml of fasting venous blood was collected and WBC (*×10^9^/L) levels were measured by Laser Flow Cytometry + Fluorescent Staining (Shenzhen Mindray Medical International Limited, Detection limit *0.99 × 10^9^/L- *100.0 × 10^9^/L); CRP(mg/mL) levels were measured by CardioPhase hsCRP (Siemens Healthcare Diagnostics Products GmbH, OQIY21, Sensitivity 0.175 mg/L, Detection limit 0.175 mg/L), IL-6(pg/mL) levels were measured by Access IL-6 (Beckman Coulter, Inc., A16370, Sensitivity 0.5 pg/mL, Detection limit 0.5 pg/mL)and procalcitonin(ng/mL) levels were measured by Access PCT (Immunotech SAS, A Beckman Coulter Company, C22594, Sensitivity 0.01 ng/mL, Detection limit 0.01 ng/mL).

### 2.4 Statistical analysis

SPSS 20.0 statistical software was used for data analysis. Missing data in the sample were imputed using the median imputation method. Measurement data is expressed in (x± s) and a t-test is used; The count data is expressed as ^[n (%)]^, using the chi-square method inspections. P<0.05 indicates statistically significant differences.

## 3 Results

### 3.1 Comparison of VAS scores at different times in the two groups

**Figure 3:**
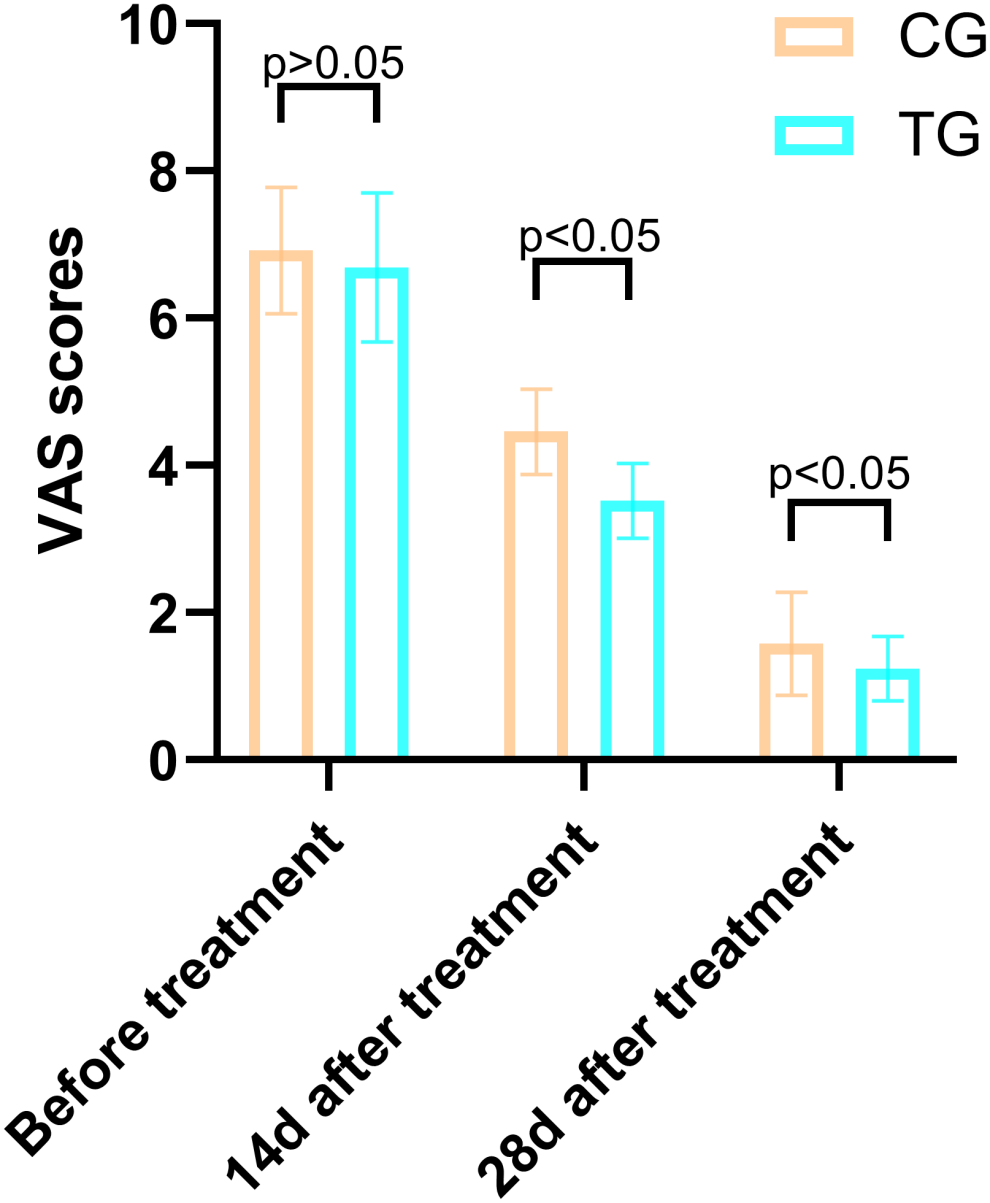
Comparison of PUSH scores between the CG and TG before treatment, 14 days, and 28 days after treatment. Significant intergroup differences emerged at 14-days and 28-days after treatment (p < 0.05). Data expressed as mean ± SD. Data expressed as mean ± SD.

Before treatment, there was no significant difference in VAS scores between the TG and the CG (6.92 ± 0.86 vs. 6.69 ± 1.01, T=0.864, P=0.392). However, after 14 days of treatment, the VAS scores in the TG were significantly lower than those in the CG (3.52 ± 0.51 vs. 4.46 ± 0.58, T=6.137, P=0.000), indicating a greater reduction in pain levels. This trend continued at 28 days, with the TG showing further improvement (1.24 ± 0.44 vs. 1.58 ± 0.70, T=2.048, P=0.046). These results suggest that ACGF treatment provides superior pain relief compared to standard wound care alone (**Figure 3**).

### 3.2 Comparison of PUSH scores between the two groups

**Figure 4:**
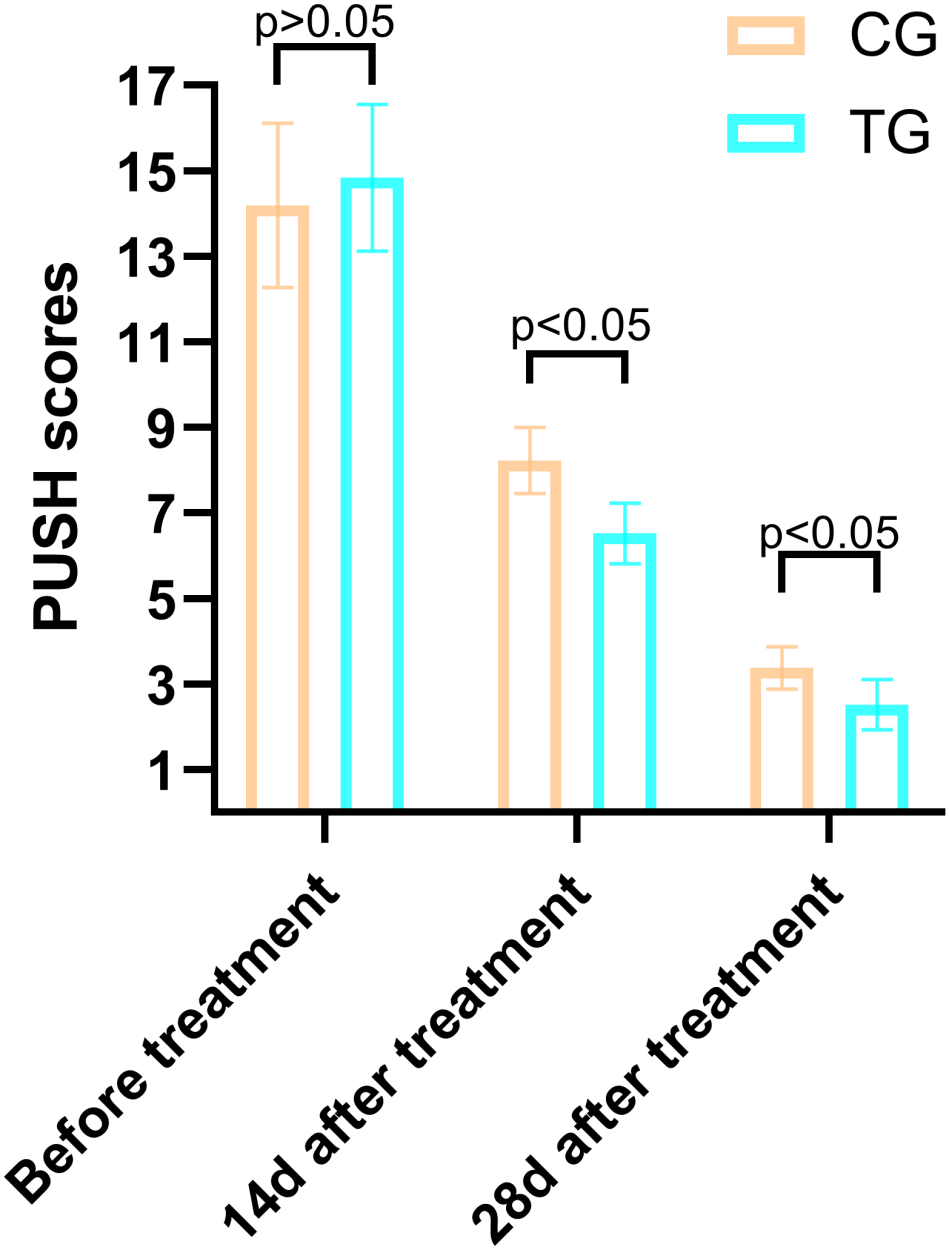
Case Report 1: **(a)** Before treatment, PUSH scores =10. **(b)** 14 days of treatment, PUSH scores =5. **(c)** 28 days of treatment, PUSH scores =2. **(d)** The pressure ulcer achieved complete healing, PUSH scores =0.

**Figure 5:**
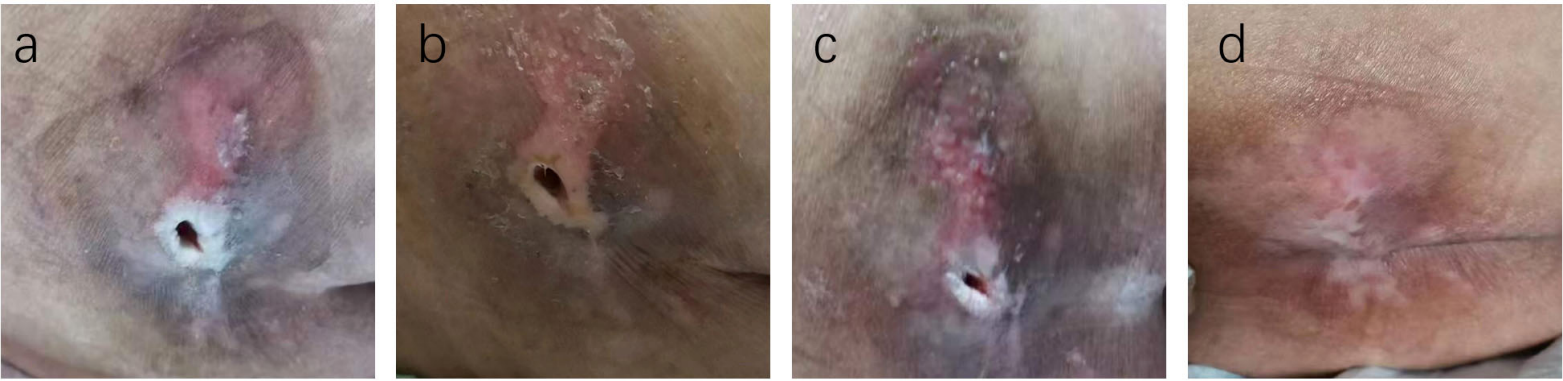
Case Report 1: **(a)** Before treatment, PUSH scores =10. **(b)** 14 days of treatment, PUSH scores =5. **(c)** 28 days of treatment, PUSH scores =2. **(d)** The pressure ulcer achieved complete healing, PUSH scores =0.

**Figure 6:**
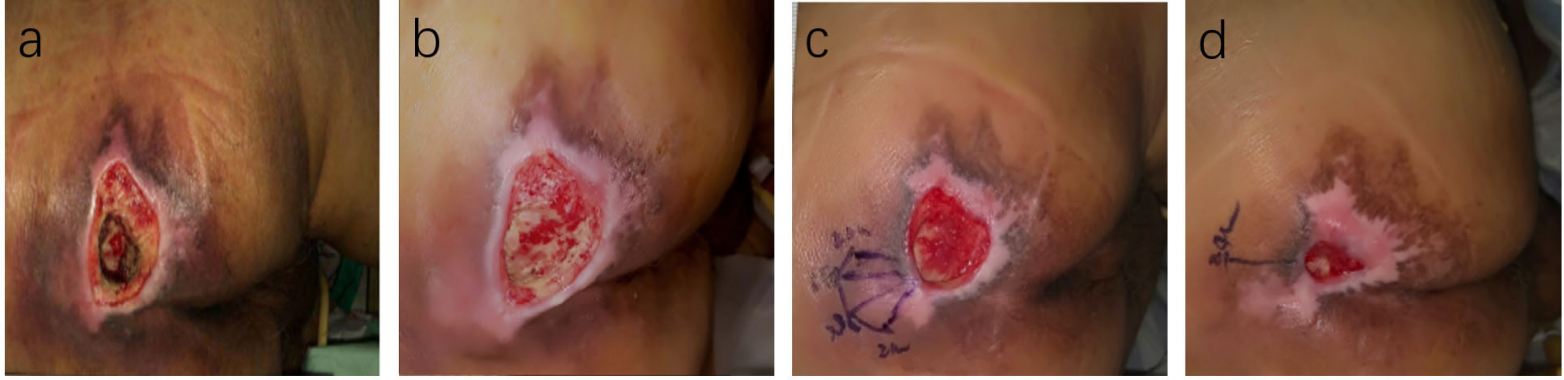
Case Report 2: **(a)** At admission, fist PUSH scores =15. **(b)** After standard treatment, the ulcer area increased, exudate increased, and the surrounding skinbecame softened, PUSH scores =17, Subsequently, ACGF treatment was administered. **(c)** 14 days of treatment, PUSH scores =9. **(d)** 28 days of treatment, PUSH scores =4.

The PUSH scores, which assess wound healing based on wound size, exudate, and tissue type, showed no significant difference between the two groups before treatment (14.84 ± 1.72 vs. 14.19 ± 1.92, T=1.266, P=0.211). However, at 14 days, the TG demonstrated a significantly greater reduction in PUSH scores compared to the CG (6.52 ± 0.71 vs. 8.23 ± 0.77, T=8.250, P=0.000). By 28 days, this difference was even more pronounced (2.52 ± 0.59 vs. 3.39 ± 0.50, T=5.695, P=0.001), indicating accelerated wound healing in the TG (**Figure 4**). Two typical cases are shown in **Figures 5**, **6**.

### 3.3 Comparison of serum levels of inflammatory factors between the two groups

**Figure 7:**
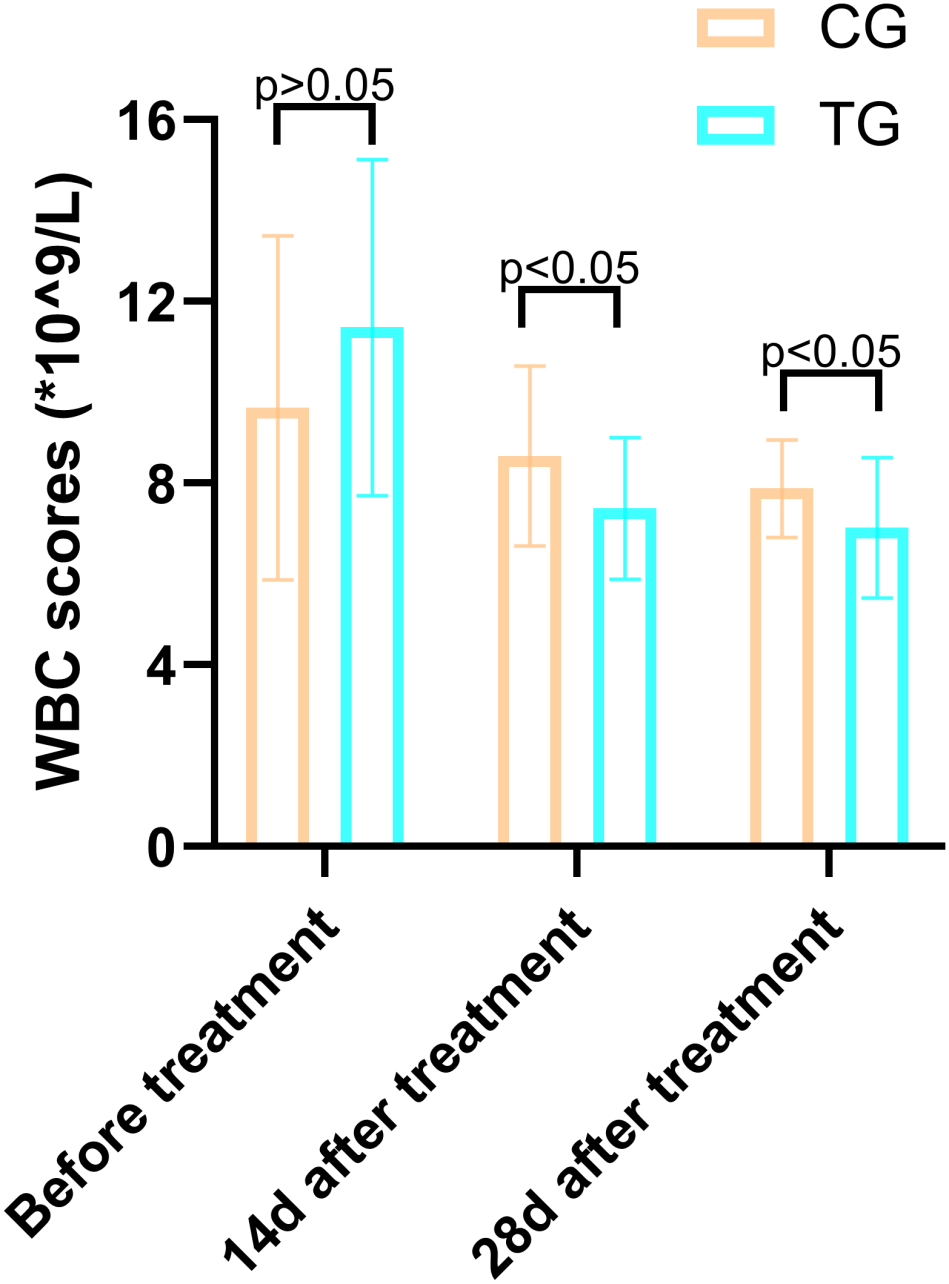
Comparison of CRP concentrations (mg/L) in CG and TG before treatment, 14 days, and 28 days after treatment. Significant intergroup difference observed at 28-day after treatment (p < 0.05). No statistical differences before treatment or 14 days (p > 0.05). Data shown as mean ± SD.

**Figure 8:**
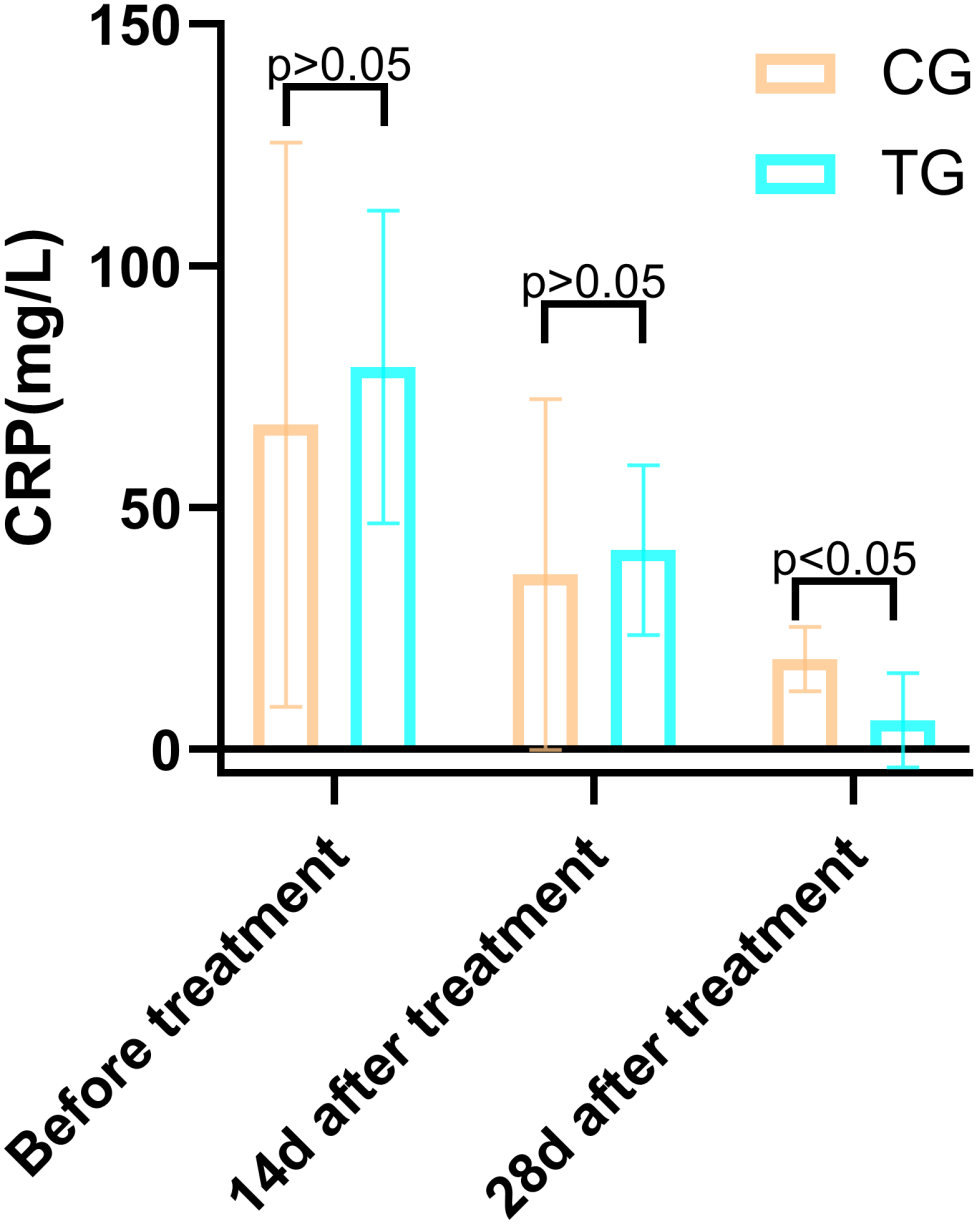
Comparison of IL-6 concentrations (pg/mL) in CG and TG before treatment, 14 days, and 28 days after treatment. Significant intergroup difference observed at 28 days after treatment (p < 0.05). No statistical differences before treatment or 14 days (p > 0.05). Data shown as mean ± SD.

**Figure 9:**
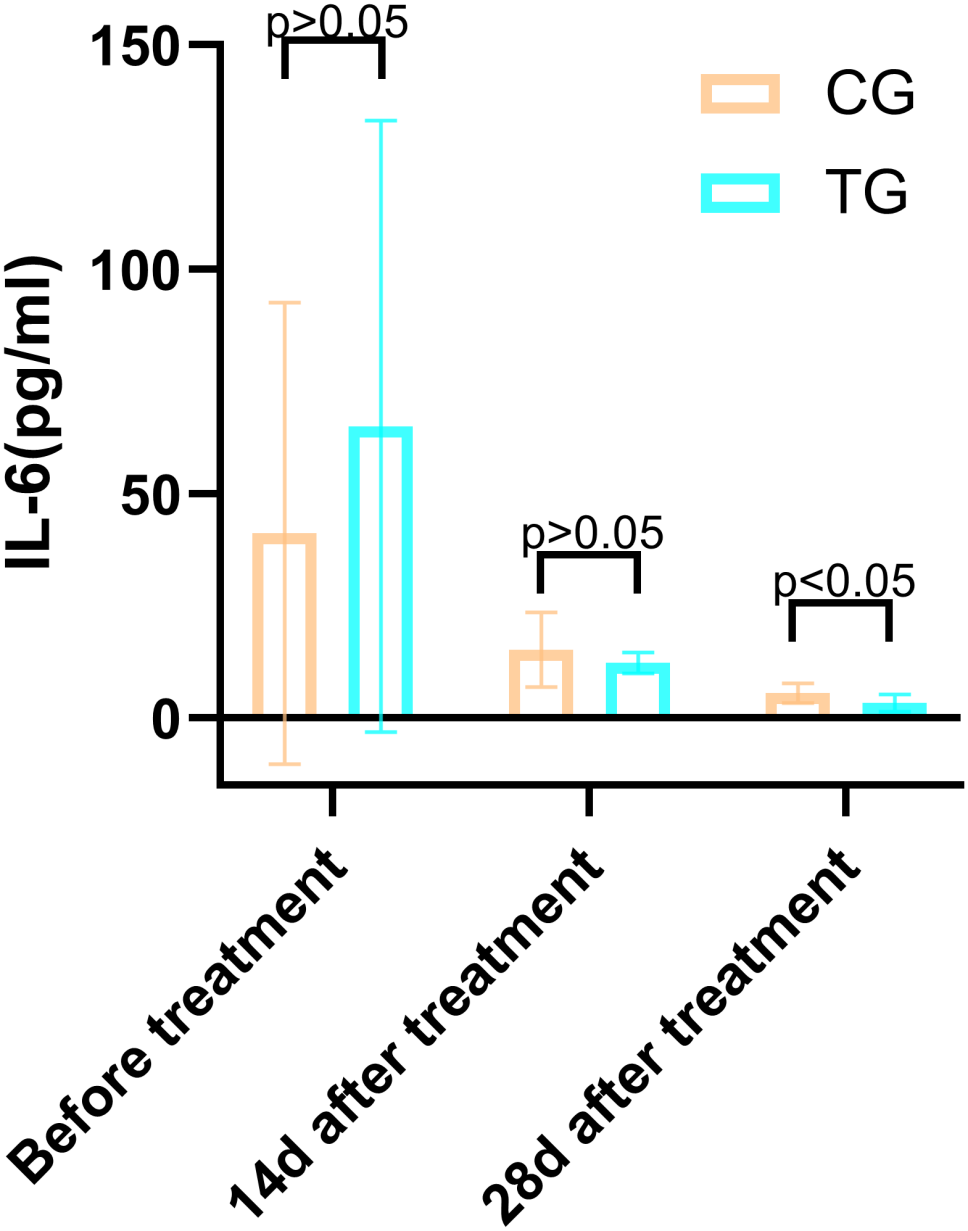
Comparison of PCT concentrations (ng/mL) in CG and TG before treatment, 14 days, and 28 days after treatment. Significant intergroup difference observed at 14 days and 28 days after treatment (p < 0.05). No statistical differences before treatment (p > 0.05). Data shown as mean ± SD.

**Figure 10:**
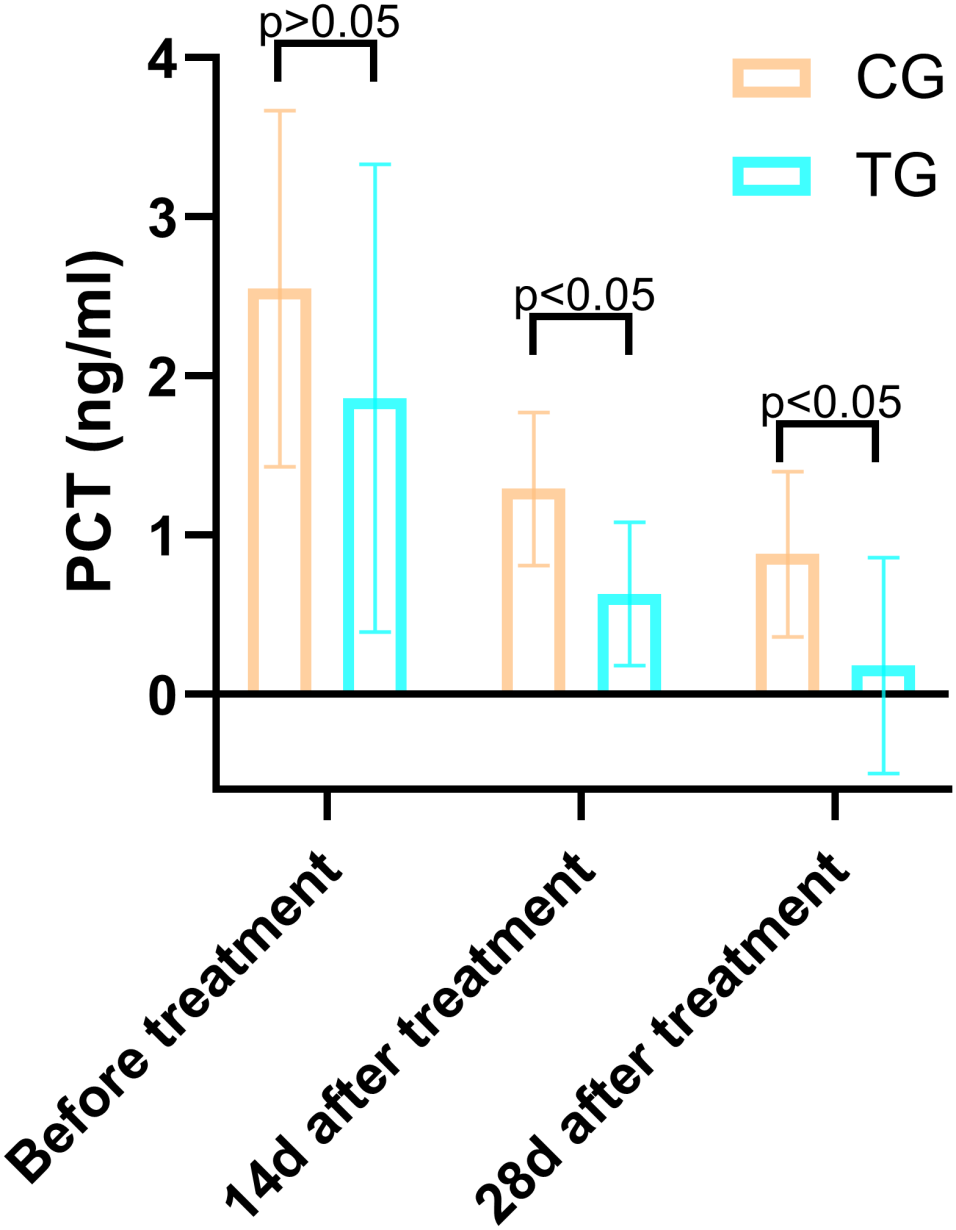
The flow diagram: A total of 55 elderly patients, aged 60 years and older, diagnosed with diabetes mellitus and suffering from grade II or higher grade were included. Four patients were excluded, and a total of 51 patients were enrolled in this study.

Inflammatory markers, including WBC, CRP, PCT, and IL-6, showed no significant differences between the two groups before treatment (P>0.05). However, at 14 days, the TG exhibited significantly lower levels of WBC (7.44 ± 1.56 vs. 8.60 ± 1.98, P=0.024) and PCT (0.63 ± 0.45 vs. 1.29 ± 0.48, P<0.01) compared to the CG. By 28 days, reductions in CRP (18.63 ± 6.62 vs. 5.93 ± 9.74, P<0.01) and IL-6 (3.35 ± 1.89 vs. 5.56 ± 2.22, P<0.01) were also observed, suggesting that ACGF treatment effectively mitigates inflammation, which is critical for wound healing (**Tables 2**–**5**, **Figures 7**–**10**).

**Table 2:** Comparison of the WBC (*×10^9^/L) scores between the two groups.

Periods	TG (n=25)	CG (n=26)	*T*	*P*
Before treatment	11.43 ± 3.70	9.66 ± 3.79	1.688	0.098
14d after treatment	7.44 ± 1.56	8.60 ± 1.98	-2.320	0.024
28d after treatment	7.02 ± 1.55	7.88 ± 1.07	-2.315	0.026

**Table 3:** Comparison of the CRP (mg/mL) between the two groups.

Periods	TG (n=25)	CG (n=26)	*T*	*P*
Before treatment	79.07 ± 32.40	67.14 ± 58.37	0.907	0.370
14d after treatment	41.17 ± 17.53	36.15 ± 36.33	0.632	0.535
28d after treatment	5.93 ± 9.74	18.63 ± 6.62	5.423	0.000

**Table 4:** Comparison of the IL-6 (pg/mL) between the two groups.

Periods	TG (n=25)	CG (n=26)	*T*	*P*
Before treatment	65.01 ± 68.12	41.21 ± 51.43	-0.565	0.164
14d after treatment	12.30 ± 2.38	15.24 ± 8.36	-1.691	0.097
28d after treatment	3.35 ± 1.89	5.56 ± 2.22	-3.830	<0.01

**Table 5:** Comparison of the PCT (ng/mL) between the two groups.

Periods	TG (n=25)	CG (n=26)	*T*	*P*
Before treatment	1.86 ± 1.47	2.55 ± 1.12	-1.900	0.063
14d after treatment	0.63 ± 0.45	1.29 ± 0.48	-5.089	0.000
28d after treatment	0.18 ± 0.68	0.88 ± 0.52	-6.651	0.000

## 4 Discussions

The treatment of diabetic pressure ulcers has always been a challenging issue in clinical practice (1, 27). Due to the frequent presence of hyperglycemia, microvascular complications, and compromised immune function in diabetic patients, the healing process of pressure ulcers is often slow and prone to infection, with limited efficacy from traditional treatment methods (28). For example, conventional debridement and moist dressing changes can alleviate some symptoms, but they are often insufficient for chronic, difficult-to-heal pressure ulcers in diabetic patients (29). Additionally, pressure ulcer treatment faces challenges such as high costs, difficulty in nursing care, and significant patient suffering (30, 31). Previous research has demonstrated that male patients typically exhibit more severe ulcer characteristics, including greater ulcer depth, higher rates of bone probe positivity, and a greater incidence of systemic infections. Furthermore, male patients are more prone to neglecting routine foot care, a behavior that can adversely affect healing outcomes (32, 33). By contrast, female patients, despite generally being older, tend to engage in more consistent self-monitoring and demonstrate better adherence to recommended treatment protocols. This adherence is reflected in their more favorable healing outcomes and a significantly higher likelihood of healing without the need for major amputation (32, 33). Recent studies, however, indicate that gender does not independently predict pressure ulcer treatment outcomes in elderly diabetic patients (32, 34). In this study, although the two groups exhibited different gender distributions, the difference was not statistically significant, which ensures the comparability of the groups.

In this study, we employed autologous concentrated growth factor (ACGF) to treat diabetic pressure ulcers and achieved significant clinical efficacy. The results showed that patients receiving ACGF treatment outperformed the control group, which received only traditional treatment, in terms of pain relief, wound healing speed, and improvement in inflammatory markers. Specifically, the VAS scores in the ACGF treatment group were significantly reduced, indicating effective pain relief, while the significant decrease in PUSH scores reflected accelerated wound healing. Furthermore, inflammatory markers (such as WBC, CRP, PCT, and IL-6) also significantly decreased after treatment, suggesting that ACGF can effectively modulate local inflammatory responses, creating favorable conditions for wound healing. The significant reduction in inflammatory markers in diabetic pressure ulcer patients treated with ACGF is primarily attributed to its rich growth factors and immunomodulatory properties (20, 35). ACGF is rich in various growth factors, such as TGF-β, PDGF, and VEGF, which not only promote cell proliferation and angiogenesis but also regulate inflammatory responses (36, 37). For example, TGF-β can inhibit the release of pro-inflammatory cytokines and reduce the infiltration of inflammatory cells (38). Additionally, the fibrin and CD34^+^ cells in ACGF form a stable scaffold, providing support for tissue repair while reducing the overexpression of inflammatory factors (39).

In the pathological process of diabetic pressure ulcers, the hyperglycemic environment and microvascular complications often lead to persistent local inflammatory responses, delaying wound healing (40). ACGF modulates the polarization of inflammatory cells, promoting the transition of M1-type macrophages to M2-type, thereby suppressing inflammation and accelerating tissue repair (41, 42). This immunomodulatory effect is particularly important in the treatment of diabetic pressure ulcers, as it effectively alleviates local inflammation and improves the wound microenvironment (43). Meanwhile, VEGF in ACGF enhances vascular permeability and promotes endothelial cell proliferation, accelerating angiogenesis (41, 44). This synergistic effect of multiple factors significantly improves the speed and quality of diabetic ulcer healing. ACGF is derived from the patient’s own blood through centrifugation, avoiding immune rejection. This autologous origin makes it safer for clinical application, reducing the risk of infection and other complications (45).

As the third-generation autologous platelet concentrate, ACGF exhibits substantial advantages over PRP and PRF. Specifically, ACGF clots have approximately 30%–50% higher platelet and associated growth factor concentrations than PRP (35). These growth factors, including PDGF, TGF-β, and VEGF, are critical bioactive molecules for wound healing. Unlike PRP, which releases growth factors rapidly, leading to a brief surge in bioavailability followed by a swift decline, ACGF’s preparation results in a denser, more physiologically structured fibrin network. This network enhances elasticity and durability, enabling sustained growth factor release for up to 30 days (46, 47). Importantly, this fibrin network also offers an optimal scaffold for cell migration, actively promoting the healing process (46, 48). Thus, ACGF therapy helps reduce treatment frequency, lower healthcare costs, and improve patient compliance, particularly for elderly patients with mobility limitations or difficulty attending frequent clinical sessions (49, 50). In summary, ACGF significantly reduces inflammatory markers, accelerates the healing of diabetic ulcers, and improves patient prognosis through multiple mechanisms. Its unique advantages in diabetic ulcer treatment make it a highly promising novel therapeutic approach.

Despite the positive outcomes of this study, there are some limitations. First, the retrospective design and relatively small sample size may limit the generalizability of the results. Second, the 28-day follow-up period is too short to assess the long-term effects of ACGF treatment, such as wound recurrence rates or the durability of healing. Future research should conduct larger-scale, prospective, randomized controlled trials with extended follow-up periods to validate these findings.

## 5 Conclusions

In conclusion, the results of this study demonstrate that ACGF, as an adjunctive treatment for elderly diabetic patients with pressure ulcers, significantly alleviates pain, accelerates wound healing, and reduces inflammatory marker levels. Through its rich growth factors and anti-inflammatory properties, ACGF effectively improves the wound healing process in diabetic patients. Although the short-term results are encouraging, further research is needed to confirm these findings, optimize treatment protocols, and evaluate long-term efficacy. ACGF represents a promising advancement in the field of regenerative medicine, offering new hope for the wound care of diabetic pressure ulcer patients.

## Data Availability

The original contributions presented in the study are included in the article/supplementary material. Further inquiries can be directed to the corresponding authors.

## References

[1] Tang J Zhang P Liu Y Hou D Chen Y Cheng L. Revolutionizing pressure ulcer regeneration: Unleashing the potential of extracellular matrix-derived temperature-sensitive injectable antioxidant hydrogel for superior stem cell therapy. Biomaterials. (2025) 314:122880. doi: 10.1016/j.biomaterials.2024.122880, PMID: 39383777

[2] Qi X Xiang Y Li Y Wang J Chen Y Lan Y. An ATP-activated spatiotemporally controlled hydrogel prodrug system for treating multidrug-resistant bacteria-infected pressure ulcers. Bioact Mater. (2024) 45:301–21. doi: 10.1016/j.bioactmat.2024.11.029, PMID: 39669125 PMC11635604

[3] McEvoy NL Kalvas LB Walsh K Curley MAQ. The identification and characterization of nurse-sensitive outcomes in acute and critical care: A systematic review. Nurs Outlook. (2025) 73:102379. doi: 10.1016/j.outlook.2025.102379, PMID: 39999613

[4] Tschannen D Anderson C. The pressure injury predictive model: A framework for hospital-acquired pressure injuries. J Clin Nurs. (2020) 29:1398–1421. doi: 10.1111/jocn.15171, PMID: 31889342

[5] Ewid M Algoblan AS Elzaki EM Muqresh MA Khalifa AR Alshargabi AM. Factors associated with glycemic control and diabetes complications in a group of Saudi patients with type 2 diabetes. Med (Baltimore). (2023) 102:e35212. doi: 10.1097/MD.0000000000035212, PMID: 37747025 PMC10519521

[6] Bhattacharya S Mishra RK. Pressure ulcers: Current understanding and newer modalities of treatment. Indian J Plast Surg. (2015) 48:4–16. doi: 10.4103/0970-0358.155260, PMID: 25991879 PMC4413488

[7] Gould L Abadir P Brem H Carter M Conner-Kerr T Davidson J. Chronic wound repair and healing in older adults: current status and future research. Wound Repair Regen. (2015) 23:1–13. doi: 10.1111/wrr.12245, PMID: 25486905 PMC4414710

[8] Luo P Huang C. Causal associations between type 2 diabetes mellitus, glycemic traits, dietary habits and the risk of pressure ulcers: univariable, bidirectional and multivariable Mendelian randomization. Front Nutr. (2024) 11:1375179. doi: 10.3389/fnut.2024.1375179, PMID: 39416647 PMC11480076

[9] Arai K Yamamoto K Suzuki T Shiko Y Kawasaki Y Mitsukawa N. Factors affecting severity of pressure ulcers: Impact of number of medications. Wound Repair Regen. (2023) 31:671–8. doi: 10.1111/wrr.13113, PMID: 37516924

[10] Naqvi SH Osundolire S Goldberg RJ Lapane KL Nunes AP. Unhealed pressure ulcers among nursing home residents with diabetes. Arch Gerontol Geriatr. (2023) 111:104969. doi: 10.1016/j.archger.2023.104969, PMID: 37004252 PMC12284829

[11] Girgis B Carvalho D Duarte JA. The effect of high-voltage monophasic pulsed current on diabetic ulcers and their potential pathophysiologic factors: A systematic review and meta-analysis. Wound Repair Regen. (2023) 31:171–86. doi: 10.1111/wrr.13063, PMID: 36507861

[12] Long L Liu W Hu C Yang L Wang Y. Construction of multifunctional wound dressings with their application in chronic wound treatment. Biomater Sci. (2022) 10:4058–76. doi: 10.1039/D2BM00620K, PMID: 35758152

[13] Norman G Shi C Westby MJ Price BL McBain AJ Dumville JC. Bacteria and bioburden and healing in complex wounds: A prognostic systematic review. Wound Repair Regen. (2021) 29:466–77. doi: 10.1111/wrr.12898, PMID: 33591630

[14] Zubair M Ahmad J. Role of growth factors and cytokines in diabetic foot ulcer healing: A detailed review. Rev Endocr Metab Disord. (2019) 20:207–17. doi: 10.1007/s11154-019-09492-1, PMID: 30937614

[15] Sabeti M Gabbay J Ai A. Endodontic surgery and platelet concentrates: A comprehensive review. Periodontol 2000. (2025) 97:308–19. doi: 10.1111/prd.12593, PMID: 39135355

[16] Li X Yang H Zhang Z Zhonghai Yan Huling Lv Yan Zhang. Concentrated growth factor exudate enhances the proliferation of human periodontal ligament cells in the presence of TNF-α. Mol Med Rep. (2019) 19:943–50. doi: 10.3892/mmr.2018.9714, PMID: 30535499 PMC6323209

[17] Xiao Q Chu W Guo J Gao J Yao W Huang M. CGF therapy: bridging androgenetic alopecia observations to psoriasis treatment via IL-17 pathway. Stem Cell Res Ther. (2024) 15:353. doi: 10.1186/s13287-024-03959-y, PMID: 39380104 PMC11462746

[18] Alshujaa B Talmac AC Altindal D Alsafadi A Ertugrul AS. Clinical and radiographic evaluation of the use of PRF, CGF, and autogenous bone in the treatment of periodontal intrabony defects: Treatment of periodontal defect by using autologous products. J Periodontol. (2024) 95:729–39. doi: 10.1002/JPER.23-0481, PMID: 37986648

[19] Perussolo J Calciolari E Dereka X Donos N. Platelet-rich plasma and plasma rich in growth factors in extra-oral wound care. Periodontol 2000. (2025) 97:320–41. doi: 10.1111/prd.12572, PMID: 39056422 PMC11808476

[20] Yahata Y Handa K Ohkura N Okamoto M Ohshima J Itoh S. Autologous concentrated growth factor mediated accelerated bone healing in root-end microsurgery: A multicenter randomized clinical trial. Regener Ther. (2023) 24:377–84. doi: 10.1016/j.reth.2023.08.006, PMID: 37711762 PMC10497983

[21] Zhang L Yuan Z Shafiq M Cai Y Wang Z Nie P. An injectable integration of autologous bioactive concentrated growth factor and gelatin methacrylate hydrogel with efficient growth factor release and 3D spatial structure for accelerated wound healing. Macromol Biosci. (2023) 23:e2200500. doi: 10.1002/mabi.202200500, PMID: 36788664

[22] Smith J Rai V. Platelet-rich plasma in diabetic foot ulcer healing: contemplating the facts. Int J Mol Sci. (2024) 25:12864. doi: 10.3390/ijms252312864, PMID: 39684575 PMC11641766

[23] Malcangi G Patano A Palmieri G Di Pede C Latini G Inchingolo AD. Maxillary sinus augmentation using autologous platelet concentrates (Platelet-rich plasma, platelet-rich fibrin, and concentrated growth factor) combined with bone graft: A systematic review. Cells. (2023) 12:1797. doi: 10.3390/cells12131797, PMID: 37443831 PMC10340512

[24] Loutati R Ben-Yehuda A Rosenberg S Rottenberg Y. Multimodal machine learning for prediction of 30-day readmission risk in elderly population. Am J Med. (2024) 137:617–28. doi: 10.1016/j.amjmed.2024.04.002, PMID: 38588939

[25] Chang CC Lai TF Chen J Liao Y Park JH Chang YJ. Age difference in the association between nutritional status and dynapenia in older adults. Nutrients. (2025) 17:734. doi: 10.3390/nu17040734, PMID: 40005061 PMC11858559

[26] Xu Y Li T Wang L Yao L Li J Tang X. Platelet-rich plasma has better results for long-term functional improvement and pain relief for lateral epicondylitis: A systematic review and meta-analysis of randomized controlled trials. Am J Sports Med. (2024) 52:2646–56. doi: 10.1177/03635465231213087, PMID: 38357713

[27] Langer G Fink A. Nutritional interventions for preventing and treating pressure ulcers. Cochrane Database Syst Rev. (2014) 2014:CD003216. doi: 10.1002/14651858.CD003216.pub2, PMID: 24919719 PMC9736772

[28] Wong D Holtom P Spellberg B. Osteomyelitis complicating sacral pressure ulcers: whether or not to treat with antibiotic therapy. Clin Infect Dis. (2019) 68:338–42. doi: 10.1093/cid/ciy559, PMID: 29986022 PMC6594415

[29] Jaul E. Assessment and management of pressure ulcers in the elderly: current strategies. Drugs Aging. (2010) 27:311–25. doi: 10.2165/11318340-000000000-00000, PMID: 20359262

[30] Demarré L Van Lancker A Van Hecke A Verhaeghe S Grypdonck M Lemey J. The cost of prevention and treatment of pressure ulcers: A systematic review. Int J Nurs Stud. (2015) 52:1754–74. doi: 10.1016/j.ijnurstu.2015.06.006, PMID: 26231383

[31] Hajhosseini B Longaker MT Gurtner GC. Pressure injury. Ann Surg. (2020) 271:671–9. doi: 10.1097/SLA.0000000000003567, PMID: 31460882

[32] Lee YJ Kim JY Dong CB Park OK. Developing risk-adjusted quality indicators for pressure ulcers in long-term care hospitals in the Republic of Korea. Int Wound J. (2019) 16 Suppl 1:43–50. doi: 10.1111/iwj.13024, PMID: 30793859 PMC7949183

[33] Fan L Wu XJ. Sex difference for the risk of amputation in diabetic patients: A systematic review and meta-analysis. PloS One. (2021) 16:e0243797. doi: 10.1371/journal.pone.0243797, PMID: 33705430 PMC7951841

[34] Lichterfeld-Kottner A Lahmann N Kottner J. Sex-specific differences in prevention and treatment of institutional-acquired pressure ulcers in hospitals and nursing homes. J Tissue Viability. (2020) 29:204–10. doi: 10.1016/j.jtv.2020.05.001, PMID: 32471633

[35] Mijiritsky E Assaf HD Peleg O Shacham M Cerroni L Mangani L. Use of PRP, PRF and CGF in periodontal regeneration and facial rejuvenation-A narrative review. Biol (Basel). (2021) 10:317. doi: 10.3390/biology10040317, PMID: 33920204 PMC8070566

[36] Chen L Cheng J Cai Y Zhang J Yin X Luan Q. Efficacy of concentrated growth factor (CGF) in the surgical treatment of oral diseases: a systematic review and meta-analysis. BMC Oral Health. (2023) 23:712. doi: 10.1186/s12903-023-03357-5, PMID: 37794381 PMC10548564

[37] Xu C Pei Y Wang Y Li W Yang L Chai A. Progress in the application of auto-concentrated growth factor (CGF) in wound repair. J Biomater Appl. (2025) 39:819–27. doi: 10.1177/08853282241305362, PMID: 39648295

[38] Masuki H Okudera T Watanebe T Suzuki M Nishiyama K Okudera H. Growth factor and pro-inflammatory cytokine contents in platelet-rich plasma (PRP), plasma rich in growth factors (PRGF), advanced platelet-rich fibrin (A-PRF), and concentrated growth factors (CGF). Int J Implant Dent. (2016) 2:19. doi: 10.1186/s40729-016-0052-4, PMID: 27747711 PMC5005757

[39] Stanca E Calabriso N Giannotti L Nitti P Damiano F Stanca BDC. Analysis of CGF biomolecules, structure and cell population: characterization of the stemness features of CGF cells and osteogenic potential. Int J Mol Sci. (2021) 22:8867. doi: 10.3390/ijms22168867, PMID: 34445573 PMC8396261

[40] Sheir MM Nasra MMA Abdallah OY. Phenytoin-loaded bioactive nanoparticles for the treatment of diabetic pressure ulcers: formulation and *in vitro*/*in vivo* evaluation. Drug Delivery Transl Res. (2022) 12:2936–49. doi: 10.1007/s13346-022-01156-z, PMID: 35403947 PMC9636106

[41] Eremenko E Ding J Kwan P Tredget EE. The biology of extracellular matrix proteins in hypertrophic scarring. Adv Wound Care (New Rochelle). (2022) 11:234–54. doi: 10.1089/wound.2020.1257, PMID: 33913776

[42] Zeng Q Zhou C Li M Qiu Y Wei X Liu H. Concentrated growth factor combined with iRoot BP Plus promotes inflamed pulp repair: an *in vitro* and *in vivo* study. BMC Oral Health. (2023) 23:225. doi: 10.1186/s12903-023-02903-5, PMID: 37076830 PMC10114309

[43] Dohan Ehrenfest DM Pinto NR Pereda A Jiménez P Corso MD Kang BS. The impact of the centrifuge characteristics and centrifugation protocols on the cells, growth factors, and fibrin architecture of a leukocyte- and platelet-rich fibrin (L-PRF) clot and membrane. Platelets. (2018) 29:171–84. doi: 10.1080/09537104.2017.1293812, PMID: 28437133

[44] Liu Y Liu Y Zeng C Li W Ke C Xu S. Concentrated growth factor promotes wound healing potential of HaCaT cells by activating the RAS signaling pathway. Front Biosci (Landmark Ed). (2022) 27:319. doi: 10.31083/j.fbl2712319, PMID: 36624939

[45] Lin J Liu J Liu Z Fu W Cai H. Effect of concentrated growth factor on wound healing, side effects, and postoperative complications following third molar surgery. J Stomatol Oral Maxillofac Surg. (2025) 126:102031. doi: 10.1016/j.jormas.2024.102031, PMID: 39236786

[46] Chen J Wan Y Lin Y Jiang H. Platelet-rich fibrin and concentrated growth factors as novel platelet concentrates for chronic hard-to-heal skin ulcers: a systematic review and Meta-analysis of randomized controlled trials. J Dermatolog Treat. (2022) 33:613–21. doi: 10.1080/09546634.2020.1773386, PMID: 32441168

[47] Elayah SA Younis H Cui H Liang X Sakran KA Alkadasi B. Alveolar ridge preservation in post-extraction sockets using concentrated growth factors: a split-mouth, randomized, controlled clinical trial. Front Endocrinol (Lausanne). (2023) 14:1163696. doi: 10.3389/fendo.2023.1163696, PMID: 37265705 PMC10231034

[48] Wang L Wan M Li Z Zhong N Liang D Ge L. A comparative study of the effects of concentrated growth factors in two different forms on osteogenesis *in vitro* . Mol Med Rep. (2019) 20:1039–48. doi: 10.3892/mmr.2019.10313, PMID: 31173196 PMC6625392

[49] Chen L Huang C Zhong Y Chen Y Zhang H Zheng Z. Multifunctional sponge scaffold loaded with concentrated growth factors for promoting wound healing. iScience. (2022) 26:105835. doi: 10.1016/j.isci.2022.105835, PMID: 36624841 PMC9823238

[50] Patton D Moore ZE Boland F Chaboyer WP Latimer SL Walker RM. Dressings and topical agents for preventing pressure ulcers. Cochrane Database Syst Rev. (2024) 12:CD009362. doi: 10.1002/14651858.CD009362, PMID: 39625073 PMC11613325

